# A cross-sectional study on knowledge and behavior regarding medication usage among guardians of left-behind children: evidence from China

**DOI:** 10.1186/s12889-023-14989-1

**Published:** 2023-01-16

**Authors:** Qiaoyue Ge, Yao Zhou, Zeyuan Sun, Xia Jiang, Lu Zhang, Chunsong Yang, Yixin Guo, Ting Luo, Yuzhi Fu, Qunfen Xu, Yuan Chen, Wei Zhou, Qian Wu, Xianghong Lian, Zhenmi Liu, Yunzhu Lin

**Affiliations:** 1 Institute of Systems Epidemiology, West China School of Public Health and West China Fourth Hospital, Sichuan University, Sichuan, Chengdu, China; 2 Department of Pharmacy, Evidence-based Pharmacy Center, West China Second University Hospital, Key Laboratory of Birth Defects and Related Diseases of Women and Children, Sichuan University, Chengdu, China; 3 Department of Child and Adolescent Psychiatry, Institute of Psychiatry Psychology and Neuroscience, King’s College London, London, UK

**Keywords:** Left-behind children, Medications, A cross-sectional study

## Abstract

**Purpose:**

The primary objective of this study was to evaluate knowledge and behavior of medication use among guardians of left-behind children (LBC) and non-left-behind children (NLBC).

**Methods:**

A cross-sectional study was conducted in Chengdu, the major city of southwestern China from May 2020 to August 2020. A logistic regression model was conducted to assess medication-related knowledge and behavior of guardians between the LBC group and NLBC group, adjusted for confounders. Stratified analysis was further performed.

**Results:**

The overall mean scores for knowledge and for behavior were 20.22 (standard deviation = 4.472) and 15.77 (standard deviation = 3.604), respectively. No significant difference was found in medication-related knowledge and behavior scores between LBC and NLBC guardians (*P* > 0.05). A significant difference was only observed after adjusting for past medical history and history of present illness (HPI).

**Conclusion:**

There was no significant difference in the awareness and behavior of medication use between guardians of LBC and NLBC in this study, having more contact with the doctor was an effective method of health education that could possibly improve their health literacy.

## Introduction

Left-behind children (LBC) are a huge and distinctive group of the population, referring to minors under the age of 16 whose parents are both migrant workers or either is a migrant worker while the other has no guardianship ability [1]. The left-behind children has long been a pressing public health challenge globally, commonly existing in low- and mid-income countries like China [2], Philippines [3] and many other countries [4] with a large proportion of workers from rural areas, where they leave their children behind for financial reasons. For example, in Philippines, approximately 27% of children (9 million) have at least one parent living abroad, while in Kyrgyzstan this figure is at least 10% (259,000 children) [5]. China, back in 2016, had an LBC population of 61 million [6]. Due to China’s rapid economic development and urbanization in recent years, a large rural-urban population shift has occurred, culminating in almost 70 million left-behind children by 2019. There are many different models of care for LBCs. Based on limited evidence, it is reported that the majority of guardians were intergenerational (grandparents), single-parent (father or mother), ascendant (relatives or neighbors), peer (older siblings) and self. Single-parent guardianship predominated, accounting for around 50–80% of all guardianships in China [7] and parents of LBCs have been encouraged by the government to have at least one parent to accompany with the children. Despite of beneficial policy aiming at encouraging both parents to work in their hometown and taking care of their children, the parents may still leave their children as LBC as a result of an improved income, uncertainty of educational opportunities and high living costs in inflow areas [8]. More than one-third of LBC is in western China, despite the population of western China is less than a tenth of that of the east [9], calling for the need for increased research attention.

Compared to NLBC, LBC suffer from higher levels of poor nutrition and physical inactivity, accident and trauma [2, 10–12]. They are also more vulnerable to sickness or develop chronic issues [13]. In addition, LBC may experience more neglect from guardians [14, 15] and receive less or inadequate care which could lead to an increased risk of health incidents [16]. As children and adolescents are at the growth and development stage, they are highly sensitive to drug reactions, forming a group with a high incidence of adverse drug reactions, including respiratory failure, brain damage, ototoxicity and even death [17, 18]. For example, the widely-used broad-spectrum antibiotics for treating severe infections (e.g., tuberculosis) in China are one of the top causes of hearing loss in children due to improper use of medication [19]. In a systematic review conducted by Smyth et al. [18], the incidence of adverse drug reactions was reported at 16.8% among all children exposed to a drug and negatively affected children’s quality of life. Meanwhile, a previous study shows that the lack of appropriate knowledge, attitudes, and practices relate to the safe use of medications [19]. Furthermore, insufficient knowledge and inappropriate behavior of medication in guardians have resulted in countless adverse drug events, especially in LBC with less care from guardians. However, the study on medication-related knowledge and behavior of guardians is quite limited, especially in southwest China, where there is little data to acknowledge the extent to which the guardians of LBC understand medication use. There is an urgent need for the region where the majority of LBCs are located or originated.

Our study aimed to evaluate knowledge and behavior of medication uses among guardians of LBC in southwestern China, the result of which would inform clinicians and guardians to pay more attention to this vulnerable population.

## Methods

### Participants

We recruited participants by convenience sampling in the pediatric outpatient clinic at the Second West China Hospital of Sichuan University from May 2020 to August 2020. All visitors were given an informed consent about our research project in the waiting area and were asked their willingness to participate in the project. All the objects included gave consent on spot, and filled out the questionnaires by themselves or under the assistance of interviewers.

We used Delphi technique and the items on questionnaire were checked for clarity and validity by 3 independent experts before its distribution to respondents. After sending the questionnaire to a small pilot group (*n* = 30), minor edits were made based on comments from the pilot group before being formally distributed to a larger sample. The reliability of Medicine knowledge and behaviors Scales was represented by a Cronbach’s α, which turned out good in pilot (Cronbach’s α = 0.792 to 0.532).

### Measurements

#### Left-behind status

LBC is defined as the children whose one or both parents migrate from where the children live and leave the children behind to live alone or with grandparents or other relatives for at least 6 months. To determine if a child belongs to LBC group, we design a cross-validation based on ‘Mean Guardian’s Age’ and ‘Child’s Age’ in the questionnaire. If the participant reported that a child belongs to non-LBC (NLBC) group, that means that the guardian is (one of) the parents, whose age is assumed to be around 20 to 50 years older than the child. Therefore, if the participant reported that the guardian’s age is over 60 years old while the child’s age is under 5, we considered this as reporting error and excluded it (*n* = 8).

#### Medicine knowledge and behaviors scales

A specific questionnaire was developed for this study, composed of 2 sections to assess the knowledge and behaviors of sampled children’s guardians in addition to the demographic Sect. (10 items): a section for guardian’s knowledge of medication for children (28 items) and a section for guardian’s behaviors on medication for children (8 items) (Table 1).

**Table 1:** Questions included in a survey of safe medication practices of guardian of children

**Knowledge Questions (True/False) **	
K1	Does the dosage of medication for children vary according to their age?
K2	Does the dosage of medication for children vary according to their weight?
K3	Can you distinguish between traditional Chinese medicine and western medicine?
K4	Are there any side effects of Chinese medicine?
K5	Can you distinguish between prescription medicine and the Over The Counter (OTC)?
K6	Do you think you need a doctor's prescription to obtain antibiotics?
K7	Is a skin test necessary before using certain antibiotics?
K8	Are you aware of adverse reactions to children's medicines commonly used at home?
K9	Do qualified drugs have adverse reactions?
K10	Do you know which medication your child is allergic to?
K11	Do antibiotics work by killing bacteria?
K12	Do antibiotics work by killing virus?
K13	Is it effective if antibiotics are not taken correctly?
K14	Do you think that the more kinds of medicine you take, the more effective it will be?
K15	Do you think that the more kinds of medicine you take, the more likely there will be adverse reactions?
K16	Do you consider antibiotics necessary for a cold or fever?
K17	Have you ever given antibiotics to your child at home on your own?
K18	Does improvement mean that you can stop taking antibiotics?
K19	Do you consult your doctor or pharmacist every time you take antibiotics?
K20	Does a common cold heal itself?
K21	Does the colour of the tablets affect the efficacy of the medicine?
K22	Does the shape of the tablet affect the efficacy of the medicine?
K23	Does drinking alcohol affect the effectiveness of a number of medicines?
K24	Is the dose of medication for children similar to that for adults?
K25	Is the bathroom a good place to store medicines?
K26	Do high temperatures and direct sunlight damage medicines?
K27	Can multiple drugs be taken together?
K28	Does child need infusion as soon as possible as long as he/she has a fever?
**Medication Behavior Questions**	
B1	Have you completely ignored the following information in the drug instructions?(indications, treatment of adverse reactions, period of validity, usage and dosage, drug interaction, batch number, precautions, contraindications, manufacturers, adverse reactions)
B2	If your child has a common illness such as a cold, diarrhoea or cough, do you usually decide whether to use medicines or not based on drug storage at home?
B3	Do you regularly check the expiry date of medicines at home?
B4	Do you check the batch numbers of your medicines at home?
B5	Do you keep store medicines in a fixed medicine cabinet out of the reach of children?
B6	What do you do if you forget to give your child their medication at the time you are administering it?
B7	Have you ever self-administered medication to your child?
B8	If your child has visits to multiple doctors, will the child take the medicine prescribed by more than one doctor?

The first section consisted of 28 closed-ended questions (yes/no) intended to assess the knowledge of children’s medications among the guardians. All of the items in the knowledge assessment have only one correct answer with each scored on a 1-point scale (1 = correct, 0 = incorrect). Higher scores represented better knowledge of children’s medications.

The second section consisted of 8 close-ended questions intended to investigate the guardians’ behaviors regarding the safety of children’s medications. The first question is a multiple-choice question, with 10 points for all items selected, and one point deducted for each missing item. All other items in the behavior assessment have only one correct answer and were scored on a 1-point scale (1 = correct, 0 = incorrect). The higher sum of scores represented superior behaviors on children’s medication. We also collected the medical record of children in our hospital and organized the past medical history and history of present illness (HPI) to reflect the frequency of guardians’ contact with doctors.

### Statistical analysis

Statistical analysis was performed with R (version 4.0.3). All data in the demographic section were categorical, the comparisons were performed using chi-square tests between the LBC group and NLBC group.

The answers to different questions regarding the medications’ knowledge/behaviors were calculated as categorical variables using a cut-off point (the median score among guardians of NLBC). A participant was categorized into good knowledge/behaviors if the sum of scores was greater than the cut-off or into low knowledge/behaviors if the sum of scores was less than the cut-off. We conducted logistic regression models with a level of knowledge/behaviors (low versus good knowledge/behaviors) as the dependent variable to assess for an independent effect of left-behind status (LBC group and NLBC group), adjusting for the kid’s age, kid’s gender, main guardian’s age, main guardian’s gender, main guardian’s occupation, residential area, family yearly income, education level of the father/mother and the frequency of the guardian meeting the child. The past medical history and HPI was also taken into account to balance the influence of health education by the health care providers that they might receive throughout their health seeking experience.

We performed a stratified analysis to control the confounding factors and to understand the stratified specific relationship between the exposure factors. We stratified by sex, age, residential area and etc. The model in the stratified analysis is the same as the model described above. *P* ≤ 0.05 was considered statistically significant for all tests. Estimated effects were presented with odds ratios (*OR*) and 95% confidence intervals (95%*CI*).

The past medical history is an overview of the child’s health before the present illness, including previous hospitalizations and any significant illness. We divided past medical history into two groups: with and without. The HPI is a chronological description of the development of the patient’s present illness from the first sign and/or symptom or from the previous encounter to the present. It includes location, quality, severity, duration, timing, associated signs and symptoms, and etc. We divided the duration of illness recorded in the HPI into four groups: less than one year, 1 ~ 2 years, 2 ~ 3years, and more than 3 years. These two categorical data will be used as confounding factors in the model.

Furthermore, we performed a sensitivity analysis in which the answers to different questions regarding the medications’ knowledge/behaviors were calculated as categorical variables using a cut-off point (the 1st quartile score or 3rd quartile score among guardians of NLBC). We conducted logistic regression models with the level of knowledge/behaviors (low versus good knowledge/behaviors) as the dependent variable to assess an independent effect of left-behind status (LBC group and NLBC group). We also conducted liner regression models with the scores of knowledge/behaviors (continuous variables) as the dependent variable. All models were adjusted for the kid’s age, kid’s gender, main guardian’s age, main guardian’s gender, main guardian’s occupation, residential area, family yearly income, education level of the father/mother and the frequency of the guardian meeting the child.

## Result

### Sociodemographic characteristics of sample subjects

Descriptive statistics regarding the sociodemographic characteristics of the survey sample are presented in Table 2. In this study, all variables were described with counts and percentages. A total of 1588 questionnaires were completed by participants of which 1451 were enrolled, resulting in a validity rate of 91.37%. 223 (15.37%) were left-behind children and 1228 (84.63%) were non-left behind children. There was no significant difference in the demographic variables between LBC and NLBC concerning the kid’s gender (*P* = 0.258), father’s education level (*P* = 0.082), mother’s education level (*P* = 0.378), main guardian’s gender (*P* = 1) and family yearly income (*P* = 0.565). Among the LBC, 81.61% were under 5 years old and 18.39% were between 6 and 11 years old. Among the NLBC, 60.91% were under 5 years old, 33.06% were between 6 and 11 years old and 6.03% were between 12 and 15 years old, which were significantly younger than LBCs (*P* < 0.001). Concerning the residential area, 84.30% LBC and 71.34% NLBC lived in suburb, with a statistically significant difference (*P* < 0.001). The result suggests that the guardian of the LBC group was older (*P* < 0.001). Compared with NLBC group, the guardians of the LBC group were more likely to work as farmers (20.36% verse 52.0%, *P* < 0.001). In addition, the LBC’s guardians meet their children more frequently than the NLBC’s guardians (*P* = 0.023) do.

**Table 2:** Demographic charateristics for participants (*N*=1451)

**Exposure**	**Total**	**Left-behind children**	**Non-left-behind children**	***P***
	***N*= 1451 (%)**	***N*= 223 (%)**	***N*= 1228 (%)**	
**Kid’s gender**				
Male	724 (49.90)	103 (46.19)	621 (50.57)	0.258
Female	727 (50.10)	120 (53.81)	607 (49.43)	
**Kid's age**				
Under 5 years	930 (64.09)	182 (81.61)	748 (60.91)	＜0.001
6-11 years	447 (30.81)	41 (18.39)	406 (33.06)	
12-15 years	74 (5.10)	0 (0)	74 (6.03)	
**Father’s level of education**				
Under junior high school	798 (54.99)	137 (61.43)	661 (53.83)	0.082
Senior high school or tecchnical secondary school	345 (23.78)	49 (21.97)	296 (24.10)	
Collage and above	308 (21.23)	37 (16.59)	271 (22.07)	
**Mother’s level of education**				
Under junior high school	826 (56.93)	135 (60.54)	691 (56.27)	0.378
Senior high school or tecchnical secondary school	343 (23.64)	45 (20.18)	298 (24.27)	
Collage and above	282 (19.43)	43 (19.28)	239 (19.46)	
**Residential area**				
Urban	387 (26.67)	35 (15.70)	352 (28.66)	＜0.001
Rural	1064 (73.33)	188 (84.30)	876 (71.34)	
**Main guardian's gender**				
Male	302 (20.81)	46 (20.63)	256 (20.85)	1
Female	1149 (79.19)	177 (79.37)	972 (79.15)	
**Main guardian's age**				
18-44 years	1098 (75.67)	0 (0)	1098 (89.41)	＜0.001
45-59 years	261 (17.99)	140 (62.78)	121 (9.85)	
Over 60 years	92 (6.34)	83 (37.22)	9 (0.73)	
**Main guardian's occupation**				
Farmer	366 (25.22)	116 (52.02)	250 (20.36)	＜0.001
Unemployed	188 (12.96)	5 (2.24)	183 (14.90)	
0thers	897 (61.82)	102 (45.74)	795 (64.74)	
**How often does the guardian meet the child?**				
Every day	1354 (93.31)	215 (96.41)	1139 (92.75)	0.023
Every month or less than above	86 (5.93)	5 (2.24)	81 (6.60)	
Every year or less than above	11 (0.76)	3 (1.35)	8 (0.65)	
**Family yearly income**				
Less than 5000 RMB	325 (22.40)	52 (23.32)	273 (22.23)	0.565
5000-20000 RMB	351 (24.19)	61 (27.35)	290 (23.62)	
20 000-50 000 RMB	284 (19.57)	36 (16.14)	248 (20.20)	
50 0000-100 000 RMB	328 (22.61)	48 (21.52)	280 (22.80)	
More than 50 0000 RMB	163 (11.23)	26 (11.66)	137 (11.16)	

All exposure are categorical variables in the raw data

### Guardian’s knowledge about the medications for the child

The overall mean knowledge scored was 20.22 (standard deviation = 4.472). Significant differences in knowledge scores were found between LBC and NLBC (*P*<0.05). After adjusting for basic confounders including kid’s age, kid’s gender, guardian’s gender, guardian’s age and residential area, we found no difference in knowledge scores between LBC and NLBC (*OR* 0.705, 95%*CI* 0.424–1.172). After additionally adjusting for confounders including main guardian’s occupation, family yearly income, education level of the father/mother and the frequency of the guardian meeting the child, we detected no statistically significant association between left-behind status and knowledge scores (*OR* 0.870, 95%*CI* 0.529–1.429).

To further examine the factors affecting it, we constructed a stratified logistic regression model. The results indicated that there was no difference in the level of guardian’s medication knowledge among LBC and NLBC in either boys (*OR* 0.794, 95%*CI* 0.468–1.347) or girls (*OR* 0.824, 95%*CI* 0.418–1.622). Moreover, when kids were younger (under 5 years old) or older (between 6 and 11 years old) neither of them was statistically significant (95%*CI* 0.456–1.486,95%*CI* 0.541–6.434). Furthermore, when the main guardian’s age was between 45 and 59 years old, no significant difference was found in the left-behind status of knowledge scores (*OR* 1.088, 95%*CI* 0.535–2.210), and when they were older than 60 years old, resulted remained similarly non-significant (*OR* 0.608, 95%*CI* 0.115–3.220). Additionally, guardians living in urban areas showed no significant effect on knowledge scores (*OR* 0.828, 95%*CI* 0.481–1.427), which was same as those living in rural areas (*OR* 1.230, 95%*CI* 0.338–4.474). There was no significant difference between children in two groups in any level of paternal education (*P*>0.05) and also non-significant in any stratum of family yearly income (*P*>0.05). More details were shown in Table 3.

**Table 3:** Drug knowledge of guardian between left-behind children and non-left-behind children (*N* = 1451)

**Variable**	***OR***	**95% confidence**		***P***
		**lower**	**upter**	
**Group**	0.870	0.529	1.429	0.582
**Kid’s gender**				
Male	0.880	0.419	1.848	0.735
Female	0.824	0.418	1.622	0.575
**Kid’s age**^a^				
Under 5 years	0.823	0.456	1.486	0.518
6-11 years	1.866	0.541	6.434	0.323
**Main guardian’s age**^a^				
45-59 years	1.088	0.535	2.210	0.816
Over 60 years	0.608	0.115	3.220	0.558
**Residential area**				
Urban	0.828	0.481	1.427	0.497
Rural	1.230	0.338	4.474	0.754
**Family yearly income**				
Less than 5000 RMB	1.200	0.386	3.734	0.753
5000-20000 RMB	0.679	0.209	2.206	0.519
20 000-50 000 RMB	0.479	0.131	1.760	0.268
50 0000-100 000 RMB	1.072	0.330	3.485	0.908
More than 50 0000 RMB	3.839	0.596	24.730	0.157
**Father’s level of education**				
Under junior high school	0.843	0.439	1.620	0.609
Senior high school or tecchnical secondary school	1.143	0.614	2.128	0.672
Collage and above	0.641	0.156	2.641	0.539
**Mother’s level of education**				
Under junior high school	0.982	0.532	1.809	0.952
Senior high school or tecchnical secondary school	0.543	0.177	1.670	0.287
Collage and above	1.032	0.210	5.078	0.969

Adjustment of kid’s age, kid’s gender, main guardian’s age, main guardian’s gender, main guardian’s occupation, residential area, family yearly income, education level of the father/mother and the frequency of the guardian meets the child

Group: LBC&NLBC

*OR* Odd ratio

^a^None left-behind children in the 12-15 years of kid's age and 18-44 years of main guardian's age subgroup

### Guardian’s behaviors about the safety of medications used for child

The behaviors of guardians were analyzed by setting questions on what they have done with children’s medicines using. The mean behaviors scores were 15.77 (standard deviation = 3.604). The difference between the left-behind and non-left-behind children was not statistically significant (*P* = 0.675). A basic logistic regression model was used. No difference was found between the two groups (*OR* 0.739, 95%*CI* 0.455–1.199). Likewise, results of the full logistic regression model did not reveal any statistically significant difference (*OR* 0.781, 95%*CI* 0.480–1.271).

Subgroup analyses stratified by kid’s age, kid’s gender, main guardian’s age, main guardian’s gender, residential area, family yearly income and education level of the father/mother showed no statistically significant difference, which is consistent with the main results. More details were shown in Table 4.

**Table 4:** Drug behavior of guardian between left-behind children and non-left-behind children (*N* = 1451)

**Variable**	***OR***	**95% confidence**		***P***
		**lower**	**upter**	
**Group**	0.781	0.480	1.271	0.320
**Kid’s gender**				
Male	0.520	0.249	1.088	0.083
Female	1.039	0.538	2.004	0.910
**Kid’s age**^a^				
Under 5 years	0.820	0.458	1.466	0.503
6-11 years	1.976	0.577	6.761	0.278
**Main guardian’s age**^a^				
45-59 years	1.190	0.577	2.456	0.637
Over 60 years	0.659	0.147	2.965	0.587
**Residential area**				
Urban	0.710	0.415	1.213	0.210
Rural	1.108	0.312	3.940	0.874
**Family yearly income**				
Less than 5000 RMB	0.729	0.283	1.874	0.511
5000-20000 RMB	0.373	0.128	1.084	0.070
20 000-50 000 RMB	1.302	0.414	4.100	0.652
50 0000-100 000 RMB	1.247	0.391	3.981	0.709
More than 50 0000 RMB	2.853	0.433	18.786	0.276
**Father’s level of education**				
Under junior high school	0.654	0.344	1.244	0.195
Senior high school or tecchnical secondary school	0.812	0.302	2.186	0.680
Collage and above	1.080	0.282	4.147	0.910
**Mother’s level of education**				
Under junior high school	0.774	0.422	1.417	0.406
Senior high school or tecchnical secondary school	0.503	0.163	1.549	0.231
Collage and above	0.916	0.201	4.179	0.910

Adjustment of kid’s age, kid’s gender, main guardian's age, main guardian’s gender, main guardian’s occupation, residential area, family yearly income, education level of the father/mother and the frequency of the guardian meets the child.

Group: LBC&NLBC

*OR* Odd ratio

^a^None left-behind children in the 12-15 years of kid's age and 18-44 years of main guardian's age subgroup

### Guardian’s health-seeking behavior

We matched our questionnaires with the medical records of each child, and found a significant difference in the history of present illness (HPI) between LBC and NLBC (*P* < 0.001). The analysis of the logistic regression model (adjusted for kid’s age, kid’s gender, guardian’s gender, guardian’s age, residential area, history of present illness and past medical history) revealed a statistically significant difference in the guardian’s knowledge (*OR* 0.424, 95%*CI* 0.206–0.875) and behaviors scores (*OR* 0.238, 95%*CI* 0.128–0.443) (see Table 5).

**Table 5:** Drug knowledge/behavior of guardian between left-behind children and non-left-behind children (*N* = 1451)

Outcomes	*OR*	95% confidence		*P*
		lower	upter	
Knowledge level	0.424	0.206	0.875	0.020
Behavior level	0.238	0.128	0.443	<0.005

Adjustment of age, area, sex, history of present illness and past medical history

### Sensitive analysis

Similar results were observed in the sensitivity analysis where we calculated other logistic regression models with a level of knowledge/behaviors (low versus good knowledge/behaviors) using a cut-off point with the 1st quartile score or 3rd quartile score among guardians of NLBC. In addition, the liner regression models with the scores of knowledge/behaviors as dependent variable showed no significant difference across left-behind status (See Table 6).

**Table 6:** Sensitivity Analysis of Drug knowledge/behavior of guardian between left-behind children and non-left-behind children (*N* = 1451)

Outcomes	cut-off point^a^	*OR*/Beta	95% confidence		*P*
			lower	upter	
Knowledge level	Median	0.870	0.529	1.429	0.582
	25% quartiles	1.395	0.797	2.443	0.244
	75% quartiles	0.934	0.543	1.609	0.806
	-^b^	0.488	-0.538	0.925	1.516
Behavior level	Median	0.781	0.480	1.271	0.320
	25% quartiles	0.727	0.418	1.264	0.258
	75% quartiles	0.827	0.477	1.435	0.501
	-^b^	-0.409	-1.271	0.454	0.353

Adjustment of kid’s age, kid’s gender, main guardian's age, main guardian’s gender, main guardian’s occupation, residential area, family yearly income, education level of the father/mother and the frequency of the guardian meets the child

^a^The medications’ knowledge/behaviors were calculated as categorical variables with different cut-off point among guardians of NLBC and performed a logistic regression mode

^b^The medications’ knowledge/behaviors were calculated as continuous variables and performed a liner regression model

## Discussion

To our knowledge, this is the first study to look into the impact of left-behind status on the guardian’s medication knowledge/behaviors of LBC. Our study found that there is no statistically significant difference in guardians’ knowledge/behaviors about the child medications between LBC and NLBC, and we also found their multiple help-seeking behavior can improve their medication knowledge and then related behaviors.

Previous studies have shown significant disparities in parenting approaches (e.g., being raised by grandparents, parents or just one parent). It is also revealed that despite of the fact that problematic physical and mental development does not appear in all LBC cases [20], they were definitely at a disadvantage to other children [21, 22]. Among these health-related behaviors, the medication use is quite essential for children in poor health. In different population group among the low- and middle-income countries, it is also vital as self-medication is essential for them to keep healthy while primary health care accessibility is extremely limited [23, 24]. According to our data, many LBC had a long medical history with multiple health seeking. Guardians gained medical knowledge from this procedure, including medication knowledge. After controlling the history of present illness and medical history, the LBC group had significantly lower knowledge and behavior scores than NLBC did, which are consistent with existing findings. It was demonstrated that frequent contact with doctors could be a useful invention for health education, improving medication knowledge/behavior and then health literacy.

This study focuses on medication use issue among the Chinese LBC, and its findings provide several implications for further research and practice. Given the importance of doctor-patient communication in the prevention and treatment, particularly among guardians of LBC when seeking help in primary care clinics, the effective communication with health staff should be taken very seriously. Firstly, Tools that can educate/grade doctor-patient communication should be developed to constantly evaluate and adjust health-related behaviors among guardians of LBC during clinic visits. Several key features that are important when discussing medications have been summarized and an assessment framework for doctor-patient communication has been established which could address and alleviate many healthcare delivery inefficiencies [25, 26]. Additionally, the government of China encourages medical staff from non-primary hospitals (higher-level hospitals) to provide Family Doctor Contract Service (FDCS) in community health service centers. Regular follow-up phone calls or in-person appointments from a family doctor can facilitate the contacts of guardians of LBC. Based on this information, health policy makers can apply the most appropriate approaches to help this vulnerable subgroup. Furthermore, this feasible procedure can also help reduce the health expenditures for the government.

There are some limitations for our study. Firstly, the present study is a cross-sectional study that can merely show the correlation instead of causality. Future studies may design to clarify this. Secondly, this study relied solely on a convenience sampling, meaning the generalization should be conduct meticulously. Thirdly, the Medicine Behaviors Scale’ Cronbach’s α value was 0.532, indicating that the scale’ reliability was a little bit poor and that it should be revised going forward.

## Conclusion

The study reported the child medication use awareness and behavior of guardians in southwestern China. There is no statistically significant difference between LBC and NLBC of guardians’ medication use awareness and behavior in this study. It also illustrated that maintaining more frequent contact with doctors is an effective method of health education, which could unconsciously improve their knowledge/behavior about the child’s medication. These findings deliver an important public health message that the communication with health staff, no matter in which levels of care, is a helpful intervention to enhance their health literacy. This should be implemented to facilitate LBC related help behaviors and meanwhile reduce the burden of their diseases.

## Data Availability

According to the data confidentiality agreement of the corresponding author, the data is not disclosed, but is stored in the secured computer, which can be obtained by the corresponding author and used for scientific research purposes only.
